# Alternative regression models to assess increase in childhood BMI

**DOI:** 10.1186/1471-2288-8-59

**Published:** 2008-09-08

**Authors:** Andreas Beyerlein, Ludwig Fahrmeir, Ulrich Mansmann, André M Toschke

**Affiliations:** 1 Ludwig-Maximilians University of Munich, Division of Pediatric Epidemiology, Institute of Social Pediatrics and Adolescent Medicine, Munich, Germany; 2 Ludwig-Maximilians University of Munich, Department of Statistics, Munich, Germany; 3 Ludwig-Maximilians University of Munich, Department of Medical Informatics, Biometry and Epidemiology (IBE), Munich, Germany; 4 King's College London, Division of Health and Social Care Research, Department of Public Health Sciences, London, UK

## Abstract

**Background:**

Body mass index (BMI) data usually have skewed distributions, for which common statistical modeling approaches such as simple linear or logistic regression have limitations.

**Methods:**

Different regression approaches to predict childhood BMI by goodness-of-fit measures and means of interpretation were compared including generalized linear models (GLMs), quantile regression and Generalized Additive Models for Location, Scale and Shape (GAMLSS). We analyzed data of 4967 children participating in the school entry health examination in Bavaria, Germany, from 2001 to 2002. TV watching, meal frequency, breastfeeding, smoking in pregnancy, maternal obesity, parental social class and weight gain in the first 2 years of life were considered as risk factors for obesity.

**Results:**

GAMLSS showed a much better fit regarding the estimation of risk factors effects on transformed and untransformed BMI data than common GLMs with respect to the generalized Akaike information criterion. In comparison with GAMLSS, quantile regression allowed for additional interpretation of prespecified distribution quantiles, such as quantiles referring to overweight or obesity. The variables TV watching, maternal BMI and weight gain in the first 2 years were directly, and meal frequency was inversely significantly associated with body composition in any model type examined. In contrast, smoking in pregnancy was not directly, and breastfeeding and parental social class were not inversely significantly associated with body composition in GLM models, but in GAMLSS and partly in quantile regression models. Risk factor specific BMI percentile curves could be estimated from GAMLSS and quantile regression models.

**Conclusion:**

GAMLSS and quantile regression seem to be more appropriate than common GLMs for risk factor modeling of BMI data.

## Background

The prevalence of childhood obesity increased dramatically during the last decades in industrialized countries [1,2]. This increase in prevalence seems rather to be due to a shift of the upper part of the body mass index (BMI) distribution than to a shift of the entire BMI distribution as for example observed in the NHANESIII survey from 1988 to 1994 [3]. This increased positive skewness could be due to exposure to obesogenic environmental determinants among a subpopulation with a high degree of susceptibility. TV watching, formula feeding, smoking in pregnancy, maternal obesity or parental social class are well known environmental, constitutional or sociodemographic risk factors [4,5]. However, it remains unknown if these factors affect the entire BMI distribution or only parts of it. A recent descriptive study reported an effect of several risk factors for childhood obesity on upper BMI percentiles, while the middle part of the BMI distribution was virtually unaffected. However, this study did not adjust for potential confounders [6].

In the literature most authors used linear or logistic regression to model effects on body mass index (BMI) measures. However, BMI data are usually positively skewed, and therefore a transformation of the response variable and/or other regression methods might be more appropriate. Possible approaches include lognormal or Box Cox power transformations of the BMI prior to linear regression modeling, gamma regression, quantile regression or GAMLSS models.

Quantile regression has been applied in various BMI-related studies [7-9]. Several risk factors for increased adult body size had different effects on specific quantiles. Comparisons between different regression models were discussed, but not quantified by model fit criteria such as Akaike Information Criterion (AIC) [10].

The aim of our study was to compare generalized linear models, GAMLSS models and quantile regression models among BMI data on 4967 preschoolers in order to identify the best approach for obesity risk factor analysis. Additionally, we aimed to assess the effect of different risk factors on the BMI distribution (change of mean, variance, skewness or kurtosis) that might have implications for preventive measures (population based approach vs. targeted approach).

## Methods

### Data

Data on 7026 children participating in the school entry health examination in Bavaria, Southern Germany, were collected between September 2001 and August 2002. Children's age ranged from 54 to 88 months. Parental questionnaires on sociodemographic, lifestyle and other risk factors for obesity were distributed together with the invitation to the compulsory school entry examination. Children's weight and height were measured in light clothing and with calibrated balances and fixed stadiometers during the examination. The study has been described in detail elsewhere [4].

Sex and age were considered as confounders, while explanatory variables with previously reported associations to childhood body composition were *a priori *considered as exposures (abbreviations in brackets). These exposure variables included maternal smoking in pregnancy (PS), amount of watching TV (TV), breast feeding (BF), daily meal frequency (MF), highest graduation of either parent (elementary/secondary/at least A-level) (PG), maternal BMI (MB) and child's weight gain from birth to 2 years of life (WG) [4,5,11]. The sample was confined to cases with complete information on these variables leaving data of 4967 children for the analyses.

### Statistical methods

Simple linear regression uses an identity link and models the relationship between a dependent variable ***Y***_*i*_, independent variables (*z*_1_, ..., *z*_*m*_) with *m *as total number of covariates included, and residuals (*ε*_1_, ..., *ε*_*n*_) for the individual *i*, *i *= 1, ..., *n*. The model can be denoted as

*y*_*i *_= *β*_0 _+ *β*_1_*z*_*i*1 _+ ... + *β*_*m*_*z*_*im *_+ *ε*_*i*_, *ε*_*i *_~ *N*(0, *σ*^2^).

*Generalized linear models *(GLM) allow a more flexible modeling [12] of the linear predictor *η*_*i *_= *g*(*μ*_*i*_) which can be denoted as

(1) *η*_*i *_= *β*_0 _+ *β*_1_*z*_*i*1 _+ ... + *β*_*m*_*z*_*im*_.

The link function *g*(.) can be specified e.g. by

• the identity link *g*(*μ*) = *μ*, resulting in the simple linear regression model,

• the log link *g*(*μ*) = log(*μ*) yielding loglinear regression,

• the Box Cox power link [13]

$$g(\lambda ,\mu )=\{\begin{array}{c} (\mu^{\lambda }-1)/\lambda ,\text{ if }\lambda \neq \text{0}\\ \log(\mu ),\text{ if }\lambda =\text{0} \end{array}$$

• or the inverse link *g*(*μ*) = *μ*^-1^.

The inverse link function is the natural link function for the normal gamma distribution and was used in this study to perform gamma regression.

One approach for model selection is the Generalized Akaike Information Criterion (GAIC)

(2) $$GAIC(c)=-2LL(\hat{\theta })+(c\times p)$$

with *c *= 2 for the 'classical' Akaike Information Criterion (AIC) [10], and *c *= log(*n*) for the Bayes Information Criterion (BIC) [14]. The GAIC includes the log likelihood

$$LL(\hat{\theta })=\log\left(L(y_{1},...,y_{n}|\hat{\theta })\right)=\sum_{i=1}^{n}\log f(y_{i}|\hat{\theta })$$

containing the relevant parameter vector $\hat{\theta }$ (e. g. *μ*) and a penalty term *c *× *p *for the number of parameters and *p *= *m *+ *f *with *f *for the extra degrees of freedom needed for special model fitting techniques (e. g. splines). A statistical model is considered as better fitting if its GAIC is smaller than the GAIC of another statistical model.

*Generalized Additive Models for Location, Scale and Shape *(GAMLSS) offer an approach to model data with consideration of *μ *as location parameter as well as *σ *as scale parameter, and the skewness parameter *ν *and the kurtosis parameter *ζ *as shape parameters. A GAMLSS model is based on independent observations *y*_*i *_for *i *= 1, ..., *n *and monotone link functions *g*_*k*_(.), relating the parameters *μ*, *σ*, *ν *and *ζ *to the *J*_*k *_explanatory variables [15,16] through semiparametric predictors. The common choice of the link functions is:

g1(μ)=μ=η1=∑j=1J1fj1(xj1)+z1'β1g2(σ)=log⁡(σ)=η2=∑j=1J2fj2(xj2)+z2'β2g3(ν)=ν=η3=∑j=1J3fj3(xj3)+z3'β3g4(ς)=log⁡(ς)=η4=∑j=1J4fj4(xj4)+z4'β4

A multiplicative rather than an additive model for *μ *can be obtained by setting *g*_1_(*μ*) = log(*μ*). Calculations with GAMLSS in this study use the Box Cox t (BCT) distribution, which is defined as

$$z=\{\begin{array}{c} 1/\sigma \nu \left({\left(y/\mu \right)}^{\nu }-1\right),\;\nu \neq 0\\ \sigma^{-1}\log(y/\mu ),\;\nu =\text{0} \end{array},$$

with *z *assumed to follow a t distribution with *ζ *degrees of freedom (*ζ *> 0). Under this assumption it is possible to perform likelihood calculations.

Additionally, cubic and penalized splines were considered to model continuous covariates [17,18]. The model selection can also be performed by GAIC because GAMLSS represents a general framework of regression models, including the class of GLMs [19]. The authors of GAMLSS used values for *c *in the range of 2 to 3 to calculate the GAIC [19].

In contrast to the above mentioned distribution based methods, quantile regression estimates conditional quantile functions. It can be used to obtain information about specific quantiles of the underlying distribution.

*Quantile regression *for the sample quantile *τ *works by minimizing

(3) $$\underset{\theta }{\min}\sum_{i=1}^{n}\rho_{\tau }(y_{i}-\eta )$$

with the so-called check function [20]

$$\rho_{\tau }(u)=u\left(\tau -I(u<0)\right)=\{\begin{array}{c} \tau \times u,\;u\geq 0\\ (\tau -1)\times u,\;u<0 \end{array}$$

In (3), the predictor in equation (1) is taken as *η *= *Q*_*τ *_with *Q*_*τ *_being the modeled *τ *quantile.

The comparison of quantile regression and generalized linear models is a major challenge due to the inapplicability of the GAIC in quantile regression. To compare GAMLSS and quantile regression, we plotted estimated values of the 90^th ^and 97^th ^BMI percentiles for weight gain in the first two years, while the other covariates were considered at their mean values (if continuous) or their modes (if categorical). We similarly calculated the estimated percentiles for each category of meal frequency, holding the other variables fixed accordingly.

All calculations were carried out with R 2.5.1 .

## Results

The overall mean of the BMI of the 4967 children was 15.34 kg/m^2 ^with a median of 15.08 kg/m^2^. The data included 2585 males (vs. 2382 females), 417 (vs. 4550) children whose mother had smoked in pregnancy, 384 children with more than 2 TV hours per day (vs. 4583 in 3 lower categories), 1197 (vs. 3770) children who had never been breastfed, 816 children with 3 daily meals at maximum (vs. 4151 with 4 or more meals), and 1466 children whose parents had only an elementary school degree or less (vs. 3501 in other categories). In addition to these categorical covariates, we considered the metric variables children's age in months with a mean of 72.86 (SD 4.77), the maternal BMI (in kg/m^2^) which ranged from 15.9 to 49.5 (mean 23.44, SD 3.99), and the children's weight gain (in kg) in the first 2 years of life, ranging from 5.5 to 15.3 (mean 9.45, SD 1.40).

**Figure 1:**
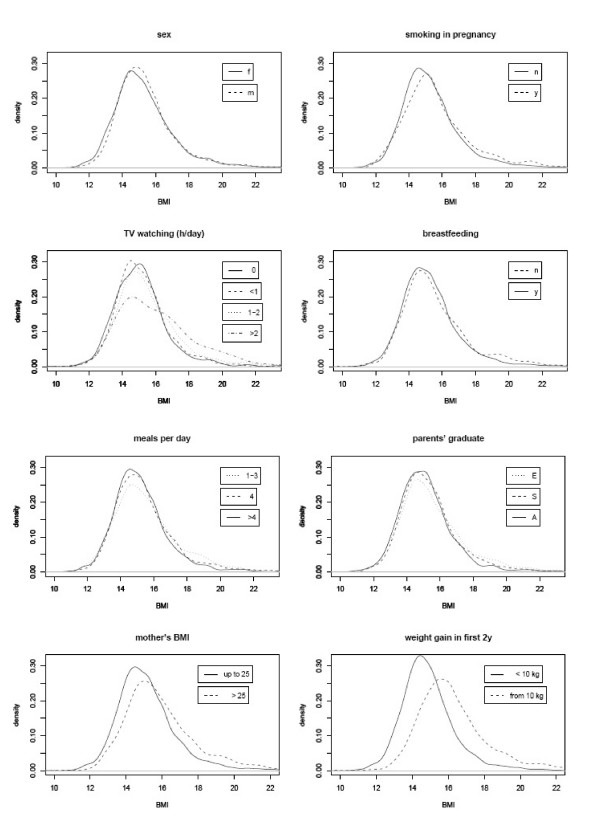
Univariate density distributions of children's BMI with regard to underlying risk factors. Maternal BMI and weight gain in the first two years were divided up into two categories. The risk factors seem to produce a slightly right-skewed distribution for exposed in comparison to non-exposed children, whereas the confounder variable sex does not.

Figure 1 shows univariate non-parametric kernel density estimates of the children's BMI distributions with regard to underlying risk factors. Maternal BMI and weight gain in the first 2 years were categorized by common cut points (Maternal BMI > 25 kg/m^2^, weight gain ≥ 10 kg [4]). When present, most risk factors seemed to increase BMI values of upper BMI regions: For example, there was a higher proportion of children with a BMI > 18 in non-breastfed compared to breastfed children, although the distribution curves of both strata were of almost identical shape for BMI values of < 18.

Simple linear models assessing the impact of certain risk factors might be limited under such varying key characteristics of the density distributions with and without underlying risk factors due to their intense assumptions.

In the multivariable regression analyses, we considered the following *a priori *defined interaction terms with reported or assumed interrelations: a) sex as confounder with every covariate except age, b) weight gain in the first 2 years with parental education [4], c) weight gain in the first 2 years with breast feeding [21] and d) maternal smoking in pregnancy with breastfeeding [22].

Full multivariable linear, loglinear, gamma and linear regression models with Box Cox power transformed BMI values included all covariates and all *a priori *defined interaction terms. The backward elimination procedure yielded models without any interaction term and without parents' graduate, maternal smoking in pregnancy or breastfeeding for all 4 GLM models,

*η *= *β*_0 _+ *β*_1_*SEX *+ *β*_2_*TV *+ *β*_3_*MF *+ *β*_4_*MF *+ *β*_4_*AGE *+ *β*_5_*MB *+ *β*_6_*WG*

with *η *= *μ *for LR, for example.

We chose *c *= 3 in equation (2) for the GAIC because this factor yielded stable and plausible results in a univariate preanalysis (data not shown). We decided not to fit the multivariable GAMLSS model by considering all covariates from the beginning and starting the fitting process due to the high computational demand of this approach. Instead, we calculated separate univariate GAMLSS models for all covariates and thereafter combined the resulting models to a multivariable model in terms of a pre-selecting forward selection procedure. During the fitting process of univariate models, we considered the strict parameter hierarchy for GAMLSS models in four steps, according to the suggestion of the GAMLSS authors [23]: first a model for *μ *should be fitted, after that for *σ*, followed by *ν *and *ζ*. If a parameter term did not reduce the GAIC(3), it was not considered for the univariate model of the respective covariate. For example, *ν *and *ζ *did not enhance the fit of the univariate model for the variable watching TV, yielding (table 1):

**Table 1:** Estimators (EST) and 95% confidence intervals (CI) of the multivariable GAMLSS model in the School Entry Health Examination Study in Bavaria, 2001–2002.

Variable	$\hat{\mu }$		log $\hat{\sigma }$		$\hat{\nu }$		log $\hat{\varsigma }$	
	EST	95% CI	EST	95% CI	EST	95% CI	EST	95% CI

Intercept	7.74	7.10, 8.38	-3.37	-3.49, -3.15	-1.41	-1.66, -1.19	1.72	-0.18, 3.62
Sex (SEX)	-0.10	-0.17, -0.03	-0.06	-0.11, -0.01	---‡	---	---	---
Watching TV (TV) *								
Up to 1 h	0.00	-0.09, 0.09	-0.03	-0.09, 0.03	---	---	---	---
1–2 h	0.08	-0.02, 0.18	0.05	-0.01, 0.11	---	---	---	---
More than 2 h	0.39	0.20, 0.58	0.21	0.12, 0.30	---	---	---	---
Breastfeeding (BF)	---‡	---	-0.08	-0.13, -0.03	---‡	---	---	---
Meal frequency (MF) †								
4/day	-0.01	-0.13, 0.11	-0.20	-0.26, -0.14	---	---	-1.48	-3.09, 0.13
5 or more/day	-0.16	-0.28, -0.04	-0.26	-0.32, -0.20	---	---	-1.94	-3.55, -0.33
Age (AGE)	0.02	0.01, 0.02	---	---	---	---	---	---
Maternal BMI (MB)	0.07 §	0.06, 0.08	0.02 §	0.02, 0.02	---	---	---	---
Weight gain in first 2 y (WG)	0.50	0.47, 0.53	0.07 §	0.06, 0.09	---	---	0.22	0.10, 0.34

* "never" as reference

† "1–3/day" as reference

‡ Parameter only significant in the respective univariate model

§ Splines used for parameter estimation

*η*_1 _= *μ *= *β*_01 _+ *β*_11_*TV*

*η*_2 _= log(*σ*) = *β*_02 _+ *β*_12_*TV*

*η*_3 _= *ν *= *β*_03_

*η*_4 _= log(*ζ*) = *β*_04_

Cubic and penalized splines up to three degrees of freedom were considered in models of the continuous covariates age, maternal BMI and weight gain in the first 2 years. Parameters that were not significant anymore in the combined multivariable model were excluded from the final multivariable model. Apart from age, increase (or decrease) in the location parameter *μ *for covariates was always associated with significant increase (or decrease) in the scale parameter *σ*.

The final multivariable GAMLSS model yielded the same significant covariates as the GLM methods using backward selection, with exception of breastfeeding for which the scale parameter *σ *was significant in the GAMLSS (tables 1 and 2). The *a priori *defined interaction terms were not significant in any considered model.

**Table 2:** Variables in the models with GLM (linear regression, lognormal regression, gamma regression, regression with Box Cox power transformation), GAMLSS, quantile regression for *τ *= 0.9 (QR 0.9) and for *τ *= 0.97 (QR 0.97) for the School Entry Health Examination Study data in Bavaria, 2001–2002.

	GLM	GAMLSS	QR 0.9	QR 0.97
Sex (SEX)	+	+	+	0
Pregnancy smoking (PS)	0	[0]	+	0
Watching TV (TV)	+	+	+	+
Breastfeeding (BF)	0	(+)	+	+
Meal frequency (MF)	+	+	+	+
Parents' graduate (PG)	0	[0]	0	0
Age (AGE)	+	+	+	0
Maternal BMI (MB)	+	+	+	+
Weight gain in first 2 y (WG)	+	+	+	+

"+" denoting significant variables, "0" non-significant variables and, in case of GAMLSS, "(+)" variables only significant for the *σ *term and " [0]" variables only significant in the univariate models.5

The fit of the multivariable GAMLSS was far better than the fit of the multivariable GLM models. The GAIC(3) of GAMLSS was 17 470, while linear regression with Box Cox Power transformation, gamma regression, loglinear regression and the simple linear regression model yielded increased GAICs with 17 955, 18 120, 18 219 and 18 616, respectively.

Apart from parental education, all considered covariates were significant in quantile regression considering the quantile *τ *= 0.9 (equals 90^th ^percentile). In quantile regression (QR) models with *τ *= 0.97 (equals 97^th ^percentile), however, only TV watching, breastfeeding, meal frequency, maternal BMI and weight gain in first two years of life were significantly associated with child's BMI. For example, the model for QR, *τ *= 0.9, was (table 3):

**Table 3:** Estimators and 95% confidence intervals (CI) of the quantile regression models with *τ *= 0.9 (QR 0.9) and *τ *= 0.97 (QR 0.97).

	QR 0.9		QR 0.97	
	Estimator	95% CI	Estimator	95% CI

Intercept	5.16	3.26, 7.06	6.33	4.61, 8.05
Sex (SEX)	-0.25	-0.47, -0.03	---	---
Pregnancy smoking (PS)	0.54	0.11, 0.97	---	---
Watching TV (TV) *				
Up to 1 h	-0.03	-0.27, 0.21	0.33	-0.20, 0.86
1–2 h	0.30	0.05, 0.55	0.68	0.23, 1.13
More than 2 h	1.31	0.80, 1.82	2.11	1.01, 3.21
Breastfeeding (BF)	-0.41	-0.72, -0.10	-0.63	-1.00, 0.26
Meal frequency (MF) †				
4/day	-0.19	-0.62, 0.24	-0.88	-1.53, -0.23
5 or more/day	-0.44	-0.01, -0.87	-1.13	-1.76, 0.50
Age (AGE)	0.03	0.01, 0.05	---	---
Maternal BMI (MB)	0.16	0.13, 0.19	0.22	0.16, 0.28
Weight gain in first 2 y (WG)	0.73	0.65, 0.81	0.87	0.75, 0.99

* "never" as reference

† "1–3/day" as reference

*η *= *β*_0 _+ *β*_1_*SEX *+ *β*_2_*PS *+ *β*_3_*TV *+ *β*_4_*BF *+ *β*_5_*MF *+ *β*_6_*AGE *+ *β*_7_*MB *+ *β*_8_*WG*

An overview on significant variables in respective models and differences across models is shown in table 2. The covariates TV watching, meal frequency, maternal BMI and weight gain in the first two years of life were significantly associated with child's BMI regardless of the method or chosen link. In contrast, parental education was not significant in any multivariable model. Its influence on offspring's BMI might sufficiently be explained by effects of the other considered covariates. An effect of breastfeeding on the BMI distribution was only detected by GAMLSS and quantile regression. Pregnancy smoking, however, was only significant in the quantile regression model of the *τ *= 0.9 quantile.

**Figure 2:**
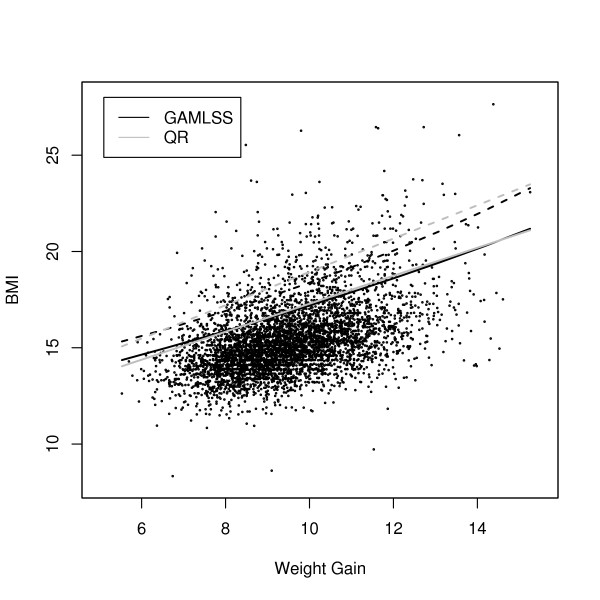
Values for the 90^th ^and 97^th ^BMI percentiles in respect to weight gain in the first two years (in kg), estimated by GAMLSS (dark lines) and quantile regression (grey lines), with fixed values for all other covariates. The dashed lines denote the estimated values for the 97^th ^percentiles for GAMLSS and quantile regression (QR), respectively. The dots represent observed values in the dataset.

In figure 2, estimated values of the 90^th ^and 97^th ^BMI percentiles from GAMLSS and quantile regression were compared for weight gain with fixed values of the other covariates. Similarly, table 4 shows percentile values estimated with both methods for different values of meal frequency. Both figure 2 and table 4 indicate that estimated values for the 90^th ^percentile obtained by GAMLSS and quantile regression were similar, while the 97^th ^percentile was slightly higher in quantile regression models. While percentile curves estimated by quantile regression were linear, those obtained by GAMLSS showed a shaped curve due to the combinations of the additional parameters *σ*, *ν *and *ζ*.

**Table 4:** Values for the 90^th ^and 97^th ^BMI percentiles (τ) estimated by GAMLSS and quantile regression (QR) in respect to meal frequency (MF), with fixed values for all other covariates.

	MF ≤ 3	MF = 4	MF ≥ 5
GAMLSS, *τ *= 0.9	17.15	16.82	16.62
QR, *τ *= 0.9	17.08	16.89	16.64
GAMLSS, *τ *= 0.97	18.39	17.96	17.83
QR, *τ *= 0.97	19.35	18.46	18.22

## Discussion and conclusion

In our study, GAMLSS showed a much better fit examining obesity risk factors compared to GLM models by GAIC. The same explanatory variables had significant associations to body composition across all GLM models, although models contained either additive (linear regression) or multiplicative components (loglinear regression, Box Cox regression and gamma regression).

In general, GAMLSS offers a flexible approach due to the large number of implemented distribution families. With GAMLSS, it is possible to assess the effect of specific parameters on the outcome variable distribution. For example, we observed that some variables did not only affect the mean, but additionally the scale of the BMI distribution. Additionally, interdependencies of considered parameters can be examined by GAMLSS. We observed that an increase (decrease) of the mean (*μ*) was mostly associated with an increase (decrease) of the scale (*σ*). The scale parameter *σ *in the distribution used (BCT) in GAMLSS is an approximative centile based coefficient of variation measure [16]. Therefore risk factors of overweight seem to affect both, the BMI itself and its variation. For example, children with a high weight gain in the first 2 years of life had higher BMI values as well as a higher coefficient of variation in BMI compared to those with a low infant weight gain. Thus, low infant weight gain might be a better predictor for underweight than is high infant weight gain for overweight. A change of the skewness term *ν*, however, did not improve the goodness of fit for modeling the skewed BMI distribution. This might be due to a sufficient consideration of skewness by a change of both parameters *μ *and *σ*.

Quantile regression allows additional interpretation, e.g. of risk factors affecting only parts of the distribution [7]. While GAMLSS models consider the entire BMI distribution, quantile regression directly examines possible associations between explanatory variables and certain predefined percentiles. Logistic regression is in principal based on a similar idea, but in case of overweight, for example, it has to deal with a big loss of information due to transformation of the continuous BMI to a binary variable. Quantile regression, in contrast, uses the whole information of the data. Furthermore, the interpretations of logistic and quantile regression differ. For example, logistic regression assesses the odds ratio for overweight in relation to certain risk factors, whereas quantile regression quantifies the linear impact of risk factors on overweight children.

In our study, the variables TV watching, maternal BMI and weight gain in the first 2 years of life were directly and meal frequency was inversely significantly associated with body composition in every examined model type. However, the strength of the associations was of different magnitude across model types (table 4).

In our study breastfeeding seemed to have a protective effect on the upper percentiles of the BMI estimated by quantile regression (e.g. -0.41 for the 90^th ^percentile, s. table 3), although generalized regression models and GAMLSS did not assess breastfeeding as being significantly associated with the mean BMI (although it was a significant predictor of *σ*). The latter is in accordance with a recent study on mean BMI and DXA derived fat mass measures [24]. Additionally, different aspects might be detected by modeling different quantiles, for example quantiles referring to underweight.

We confined our sample to cases with complete information in all variables. Since underreporting with respect to pregnancy smoking and high values of maternal BMI is well-known, this might have led to underestimation of the effects of the corresponding covariates on childhood BMI. However, such an underestimation is likely to similarly affect all examined statistical approaches and therefore be of minor relevance for assessment of the appropriate approach. It might be of interest, however, to compare how sensitive the statistical models are to several methods of missing data imputation such as multiple imputation. However, this question leads deeply into other statistical methodology and is therefore beyond the scope of our study.

GAMLSS and quantile regression have recently been compared, along with many other methods, in a WHO study to identify standard reference values for child growth [25]. Four out of five construction methods taken under further examination were GAMLSS methods with different distribution functions: Box Cox t (like in this study), Box Cox power exponential [26], Box Cox normal [27] and Johnson's SU (sinh^-1 ^normal) [28]. The other considered method used modulus-exponential-normal distribution [29]. The authors finally calculated reference values by GAMLSS with Box Cox power exponential distribution, using AIC and GAIC(3) in parallel for model selection [30]. This indicates that GAMLSS is a very appropriate method for constructing reference curves which are based on estimated percentile curves.

In our study, a comparison of GAMLSS and quantile regression by estimated values of the 90^th ^and 97^th ^percentiles with respect to certain covariates (weight gain and meal frequency) showed similar results for both methods at the 90^th ^percentile, while the estimated 97^th ^percentile was slightly higher in the quantile regression model. Since implementation of percentile curves is existent only for univariate models in the *gamlss *package, some computational effort was necessary to gain the respective GAMLSS curves with fixed effects of other covariates. Furthermore, it might be worthwhile to consider nonlinear quantile regression (20) in future studies.

The statistical model that should be used, largely depends on the observed data and on the aim of the study. GAMLSS models provide exact modeling of continuous outcomes, e.g. for the calculation of standard reference values. While GLMs provide helpful information on mean response changes, GAMLSS additionally provides information on distribution parameters like scale or skewness. On the other hand, quantile regression can be used to model specific parts of the BMI distribution such as the 90^th ^or 97^th ^percentile and should be preferred to logistic regression if the original scale of the outcome variable was continuous and a GLM or GAMLSS cannot answer the research question.

## Competing interests

The authors declare that they have no competing interests.

## Authors' contributions

The authors' responsibilities were as follows: AB (guarantor) did the statistical analysis with help by LF and wrote the first draft of the manuscript. AMT, LF and UM reviewed and critiqued the manuscript and made substantial intellectual contributions to subsequent drafts. AB and AMT had the idea for the study and wrote the final draft together.

## Pre-publication history

The pre-publication history for this paper can be accessed here:


